# Structural and Functional Aspects of G-Quadruplex Aptamers Which Bind a Broad Range of Influenza A Viruses

**DOI:** 10.3390/biom10010119

**Published:** 2020-01-10

**Authors:** Anastasia A. Novoseltseva, Nikita M. Ivanov, Roman A. Novikov, Yaroslav V. Tkachev, Dmitry A. Bunin, Alexandra S. Gambaryan, Vadim N. Tashlitsky, Alexander M. Arutyunyan, Alexey M. Kopylov, Elena G. Zavyalova

**Affiliations:** 1 Chemistry Department, Lomonosov Moscow State University, 119991 Moscow, Russia; nm-ivanov@live.com (N.M.I.); bunin_dm@mail.ru (D.A.B.); tashlitsky@belozersky.msu.ru (V.N.T.); kopylov.alex@gmail.com (A.M.K.); zlenka2006@gmail.com (E.G.Z.); 2 Engelhardt Institute of Molecular Biology RAS, 119991 Moscow, Russia; novikovfff@bk.ru (R.A.N.);; 3 Chumakov Federal Scientific Centre for Research and Development of Immune and Biological Products RAS, 108819 Moscow, Russia; al.gambaryan@gmail.com; 4 Belozersky Research Institute of Physical Chemical Biology, Lomonosov Moscow State University, 119991 Moscow, Russia

**Keywords:** DNA aptamer, G-quadruplex, influenza virus, hemagglutinin, affinity, structure–activity relationship

## Abstract

An aptamer is a synthetic oligonucleotide with a unique spatial structure that provides specific binding to a target. To date, several aptamers to hemagglutinin of the influenza A virus have been described, which vary in affinity and strain specificity. Among them, the DNA aptamer RHA0385 is able to recognize influenza hemagglutinins with highly variable sequences. In this paper, the structure of RHA0385 was studied by circular dichroism spectroscopy, nuclear magnetic resonance, and size-exclusion chromatography, demonstrating the formation of a parallel G-quadruplex structure. Three derivatives of RHA0385 were designed in order to determine the contribution of the major loop to affinity. Shortening of the major loop from seven to three nucleotides led to stabilization of the scaffold. The affinities of the derivatives were studied by surface plasmon resonance and an enzyme-linked aptamer assay on recombinant hemagglutinins and viral particles, respectively. The alterations in the loop affected the binding to influenza hemagglutinin, but did not abolish it. Contrary to aptamer RHA0385, two of the designed aptamers were shown to be conformationally homogeneous, retaining high affinities and broad binding abilities for both recombinant hemagglutinins and whole influenza A viruses.

## 1. Introduction

An aptamer is a synthetic oligonucleotide which exhibits high specificity to a target: a small molecule [1], a protein [2], a virus or a cell [3,4]. Aptamers are produced using a SELEX (Systematic Evolution of Ligands by EXponential enrichment) procedure in which approximately 10^14^ oligonucleotides compete for binding to the target, and the oligonucleotides with the highest affinities are selected [5,6,7,8,9,10,11,12]. Similar to antibodies, aptamers are molecular-recognizing elements that can be used as sensors for the influenza virus [13,14,15,16,17].

Influenza hemagglutinin is one of the major viral envelope glycoproteins, which is responsible for the attachment and penetration of the influenza virus into the host cell [18]. The following abbreviations are used further: HA for hemagglutinin in general, HA1 for the first subunit of HA, which binds sialic acids on the cell surface, and Hn (1 ≤ n ≤ 18 for influenza A) for different subtypes of hemagglutinin based on antigenic properties [19,20]. It is known that sequences of HAs from different strains differ drastically; however, the spatial structure is highly conserved [21]. Several monoclonal antibodies to hemagglutinin with broad strain-specificity were previously identified [22], and aptamers with similar properties were found recently [23,24].

To date, several aptamers to HA of influenza A virus have been described that vary in affinity and strain specificity [15], including aptamers with a G-quadruplex structure [25]. The RHA0385 aptamer was obtained to the HA1 subunit of the A/Anhui/1/2005 (H5N1) virus [23]. The activity of RHA0385 to HAs of different strains was described previously [23,24]: A/California/04/2009 (H1N1), A/Brisbane/59/2007 (H1N1), A/Georgia/20/2006 (H1N1), A/Puerto-Rico/8/1934 (H1N1), A/New Caledonia/20/1999 (H1N1), A/Aichi/2/1968 (H3N2), A/Brisbane/10/2007 (H3N2), A/Wisconsin/67/2005 (H3N2), A/Moscow/10/1999 (H3N2), A/Mississippi/1/1985 (H3N2), A/duck/Buryatia/664/1988 (H3N2), A/England/42/1972 (H3N2), A/Anhui/1/2005 (H5N1), A/JWEye/HK/1038/06 (H5N1), A/chicken/Kurgan/3654at/2005 (H5N1), A/Vietnam/1203/2004-PR8/CDC-R (H5N1), A/tern/South Africa/1/1961 (H5N3), A/mallard/Sweden/91/2002 (H7N9), A/Primorie/3631/2002 (H9N2), A/duck/Primorie/3691/2002 (H12N2). In the case of the A/chicken/Kurgan/3654at/2005 (H5N1) and A/duck/Primorie/3631/2002 (H9N2) strains, the overall amino acid sequence identity for HA is less than 50%; nevertheless, aptamer RHA0385 binds both viruses with similar affinity [24]. The causes for this broad recognition ability of the RHA0385 aptamer remain to be determined.

The structure of the RHA0385 aptamer is the main factor underlying its intriguing recognizing abilities, because the function of aptamer relies on its structure [2,26]. The starting point of our work was the hypothesis that the aptamer as a molecular-recognizing element could be regarded as a rigid scaffold with flexible loops. For example, in the thrombin-binding aptamer TBA, the antiparallel G-quadruplex core supplies the proper conformation of two lateral TT-loops, which bind thrombin. Substitutions and modifications in the loops affect the affinity [27,28,29,30,31]. Thus, altering the loops of RHA0385 while retaining the scaffold could be a robust approach for manipulating affinity.

The spatial structure of RHA0385 has not been determined, though it contains an apparent G-quadruplex pattern [23]. Here, we established the G-quadruplex scaffold of the aptamer and modified one of the loops in order to manipulate the affinity and strain specificity of the aptamer.

## 2. Materials and Methods 

### 2.1. Reagents

Glutaric aldehyde, sodium azide, KCl, NaCl, KH_2_PO_4_, K_2_HPO_4_, acetic acid, sodium acetate, DMSO, acetonitrile, 3,3′,5,5′-tetramethylbenzidine, Tween-20, and other chemicals were purchased from Helicon, Russia. A 3% hydrogen peroxide solution was purchased from Rosbio, Russia. All solutions were prepared in deionized H_2_O (obtained with MilliPore equipment from Merck, Darmstadt, Germany), and D_2_O (Helicon, Moscow, Russia) was also used for NMR experiments.

PBSK buffer was prepared immediately prior to experiments from PBS tablets (MP Biomedicals, Solon, OH, USA) with additional KCl to achieve 10 mM overall potassium ions concentration. PBST buffer was prepared from PBS tablets with the addition of 1/10,000 (*v*/*v*) Tween-20. PBSKT was prepared from PBSK with addition of 1/10,000 (*v*/*v*) Tween-20. Acetate buffer pH 5.0 was prepared immediately before experiments from 10 mM sodium acetate solution adjusted to the correct pH with acetic acid. Streptavidin horseradish peroxidase conjugate (Str-HRP) working buffer was prepared from PBS with 0.05% (*w*/*v*) bovine serum albumin and 1/10,000 (*v*/*v*) Tween-20. All buffers were filtered through a membrane with 0.22 µm pores (MilliPore, Darmstadt, Germany) and degassed.

DNA aptamers and their biotinylated derivatives were chemically synthesized and purified by Evrogen, Russia or Syntol, Russia. All biotinylated aptamers contained biotin at the 5′-end. The sequences of aptamers were as follows:

RHA0385-TTGGGGTTATTTTGGGAGGGCGGGGGTT (28 nt),
G7-TTATTAA-TTGGGGTTATTAAGGGAGGGCGGGGGTT (28 nt),
G7-TAAGAA-TTGGGGTAAGAAGGGAGGGCGGGGGTT (27 nt),
G7-TTA-TTGGGGTTAGGGAGGGCGGGGGTT (24 nt).

Recombinant HA1 subunits H1 (ab69741), H3 (ab69749), H5 (ab190125), H7 (ab190421), and H9 (ab67740) were purchased from Abcam, Burlingame, CA, USA. For two of the recombinant HAs, the viral strains are provided (A/Vietnam/1203/2004 (H5N1) and A/Anhui/1/2013 (H7N9)). Fetuin from fetal bovine serum (Sigma-Aldrich, St. Louis, MO, USA) and Str-HRP (GE Healthcare, Pittsburgh, PA, USA) were used. 

Influenza A viruses (A/England/42/1972 (H3N2), A/chicken/Kurgan/3654at/2005 (H5N1), A/mallard/Sweden/91/2002 (H7N9), A/duck/Primorie/3628/2002 (H9N2)), the control influenza B virus (B/Victoria/2/1987), allantoic fluid, and chicken erythrocytes were provided by the Chumakov Federal Scientific Centre for Research and Development of Immune and Biological Products of the Russian Academy of Sciences. All viruses were inactivated by the addition of 0.05% (*v*/*v*) glutaric aldehyde, and 0.03% (w/w) sodium azide was added as a preservative.

### 2.2. Aptamer Preparation

Immediately before each circular dichroism (CD), size-exclusion high performance liquid chromatography (SE-HPLC), surface plasmon resonance (SPR), and enzyme-linked aptamer assay (ELAA) experiment, a 2 μM aptamer solution in PBSK was prepared and annealed (heated at 95 °C for 5 min, then cooled to room temperature) to allow the tertiary structure assembly. For SE-HPLC experiments, solutions with higher aptamer concentrations (200 and 400 μM) were also prepared in the same way. The exact aptamer concentrations in each sample were calculated for each sample from the absorbance at 260 nm, 20 °C. The extinction coefficients used were calculated from the nearest-neighbor model [32]. 

### 2.3. Circular Dichroism Spectroscopy (CD)

CD spectra of aptamers in PBSK (10 mM potassium cations and 140 mM sodium cations) were obtained with a Chirascan spectrometer (Applied Photophysics Ltd., Leatherhead, Surrey, UK) equipped with a temperature controller. The PBSK spectrum was used as a baseline. The aptamers were tested at 2 µM strand concentration. The initial spectra were recorded at 25 °C. Melting and annealing curves were obtained by heating the sample from 20 to 85 °C at a rate of 0.5 °C/min. The melting and then annealing processes were followed by recording the CD spectra at wavelengths of 220–360 nm. Each spectrum was baseline corrected and divided by the aptamer concentration to generate molar circular dichroism Δε:

Δε = (ε(aptamer) − ε(PBSK))/C(aptamer).(1)

To obtain melting and annealing curves and calculate thermodynamic parameters, the positive maximum of the CD spectra (263 nm) was followed at a rate 0.5 °C/min with the spectra registration at each 2.5 min [33]. Melting curves were approximated with sigmoidal curves of the Boltzmann model for obtaining melting temperatures. 

In the approach of the cooperative and two-state process of aptamer melting, there is an equilibrium between two conformations: folded and unfolded (or denatured) [34]:

folded fraction = (ε_T_ − ε_f_)/(ε_d_ − εf), (2)

where ε_T_ is the molar CD at 263 nm at temperature T, ε_f_ is the molar CD at 263 nm of the folded structure (initial plateau), and ε_d_ is the molar CD at 263 nm of the denatured structure (final plateau). 

The equilibrium constant of the folding process, K, can be evaluated as a function of temperature:

K = [folded]/[denatured] = (folded fraction)/(1 − folded fraction).(3)

Using a van’t Hoff approach (a plot of lnK vs. 1/T), the enthalpic ΔH° and entropic ΔS° contributions to the Gibbs free energy ΔG° of the aptamer structure denaturation can also be determined as follows [35]:

lnK = ΔH°/RT − ΔS°/R ΔG° = −RTlnK = ΔH° − TΔS°.(4)

The Gibbs free energy ΔG°_298_ was calculated for the temperature 25 °C. All thermodynamic parameters refer to the folding process, e.g., a negative value of ΔG means that folding prevails over unfolding and the aptamer structure is stable under these conditions.

### 2.4. Nuclear Magnetic Resonance (NMR)

Aptamer samples were prepared in NMR tube at a concentration range from 12 to 510 mM in 0.55 mL H_2_O + D_2_O (10%) PBSK buffer solution and annealed by heating at 95 °C for 5 min and subsequent immediately cooling to 0 °C to ensure appropriate folding. The exact aptamers concentrations were as follows: 240 and 12 μM for RHA0385, 510 and 25 μM for G7-TTATTAA, 430 and 55 μM for G7-TAAGAA, and 180 and 36 μM for G7-TTA. NMR spectra were recorded with ‘Bruker AVANCE III HD 300’ and ‘Bruker AVANCE III 400’ spectrometers (300.1 and 400.1 for 1H, respectively) from Bruker, USA. 1H chemical shifts were referenced relative to external sodium 2,2-dimethyl-2-silapentane-5-sulfonate (DSS). 1D proton (1H) spectra of samples in H_2_O+D_2_O were recorded at +5 to +7 °C using pulsed-field gradient ‘WATERGATE W5’ pulse sequence (‘zggpw5 from Bruker library) for complete H_2_O-signal suppression. A relaxation delay of ~1 s and an acquisition time of ~1.35 sec were used for all experiments. The NMR data were processed using ‘Bruker TopSpin 3.6’ software (Bruker, Billerica, MA, USA). For a precise baseline correction of imino-protons region of spectra to suppress ‘mathematically’ a wide oligomeric hump, ‘multipoint baseline correction’ algorithm was applied using ‘MestReNova’ software (Mestrelab, Santiago de Compostela, Spain).

### 2.5. Size-Exclusion High Performance Liquid Chromatography (SE-HPLC)

SE-HPLC was conducted with an Agilent 1200 HPLC system with autosampler and diode array detector (Agilent, Santa Clara, CA, USA) at 25 °C. The HPLC column TSKgel G2000SWXL (Tosoh Bioscience, South San Francisco, CA, USA) was intended for the separation of 5–150 kDa proteins. The parameters of the column were follows: 30 cm length, 0.78 cm diameter, 5 µm diameter of particles, 12.5 nm mean pore diameter. The separation was performed at the flow rate of 0.5 mL/min. The mobile phase consisted of water and acetonitrile in a 9:1 *v*/*v* ratio, supplemented with potassium phosphate buffer (60 mM KH_2_PO_4_ and 140 mM K_2_HPO_4_, pH 6.85). Absorption at 260 nm was registered with a 10 nm bandwidth. The calibration of the column and the experiments were performed as described previously [36,37].

### 2.6. Surface Plasmon Resonance (SPR)

SPR experiments were conducted with a ProteOn XPR36 system (Bio-Rad, Hercules, CA, USA) at 25 °C. Solutions of 10 μg/mL recombinant HAs in acetate buffer pH 5.0 were used for immobilization by amine coupling on a GLM chip using the ProteOn Amine Coupling Kit. One channel of the chip was left without any protein, so it could be used as reference. Aptamer solutions in PBSK with 25, 50, 100, and 200 nM aptamer concentrations were injected at a flow rate of 100 µL/min for 200 s. The dissociation phase was performed for 600 s in PBSK at a flow rate of 100 µL/min. To regenerate the protein on the chip surface, the bound aptamers were completely removed by injecting PBS with 300 mM NaCl and 0.01% Tween-20. Values of the kinetic constants of complex association (k_on_) and dissociation (k_off_) were determined using the exponential approximations of the sensorgrams [38]. Apparent dissociation constants aK_D_ were calculated from the equation aK_D_ = k_on_/k_off_.

### 2.7. Hemagglutination Tests for Influenza A Virus Characterization

The standard protocol was used (VIRAPUR, San Diego, CA, USA, http://www.virapur.com/protocols/HA%20Protocol.pdf). V-bottom 96-well plates were from Greiner, Austria. Virus loads in viral particle per mL (VP/mL) were estimated from hemagglutination units (HAU/mL) based on correlations published previously [39].

### 2.8. Enzyme-Linked Aptamer Assay (ELAA)

All ELAA experiments were performed at room temperature as described previously [24]. Approximately 100 μL of a 10 μM fetuin solution in 140 mM NaCl was adsorbed over 24 h to the wells of a polystyrene 96-well plate for ELISA (Medpolimer, Saint Petersburg, Russia). The solution was removed, and the wells were washed three times with 200 μL distilled water. Solutions of influenza viruses were diluted to 128 HAU with 140 mM NaCl. Approximately 50 μL of these solutions were added to each well of the fetuin plate. After incubation for 24 h, the wells were washed 5 times with 100 μL PBST. Approximately 50 μL of biotinylated aptamer (with 1/10,000 (*v*/*v*) Tween-20 added after preformation) in PBSKT were added in serial 2-fold dilutions in the concentration range of 0–1000 nM. After incubating for one hour, the wells were washed 5 times with 100 μL PBST. Approximately 50 μL of 1/1000 (*v*/*v*) solution of streptavidin horseradish peroxidase conjugate in Str-HRP working buffer was added. After incubating for one hour, the wells were washed 5 times with 100 μL PBST. Then, 50 μL of substrate solution (0.05 mg/mL 3,3′,5,5′-tetramethylbenzidine, 0.033% H_2_O_2_ in 50 mM acetate buffer pH 4.5) was added. The peroxidase reaction was carried out for 30 min and stopped by the addition of 100 μL 5% (*v*/*v*) H_2_SO_4_. Absorption at a wavelength of 450 nm was measured with a TECAN Spark 10M microplate reader (Tecan Group Ltd., Männedorf, Switzerland). Curves were processed using Origin software (OriginLab, Northampton, MA, USA) and the apparent dissociation constants were determined by approximation with the exponential decay function.

### 2.9. Phylogenetic Analysis

Phylogenetic tree of hemagglutinins from influenza A viruses and the influenza B virus was calculated by Phylogeny.fr software [40]. The reliability of each internal branch was tested by the bootstrap method with 100 resamplings. The amino acid sequences of HA1 subunits were obtained from databases; in the cases with mutations, the most abundant record was chosen. The strains and GenBank/Protein databases codes for each sequence are as follows: A/England/42/1972 (H3N2) (EF626613.1/ABQ58930.1), A/chicken/Kurgan/3654at/2005 (H5N1) (HQ724523.1/ADU02098.1), A/VietNam/1203/2004(H5N1) (AY818135.1/AAW80717.1), A/teal/Primorie/3631/02 (H9N2) (DQ787802.1/ABI17550.1). The A/mallard/Sweden/91/2002 (H7N9) and B/Victoria/2/1987 HA1 sequences were taken from the Uniprot database (Q3KRH9 and PP22092, respectively), and A/Anhui/1/2013 (H7N9) was taken from the GISAID database (EPI439507).

## 3. Results

### 3.1. Putative Structure of the Initial Aptamer and Design of Its Derivatives

**Figure 1:**
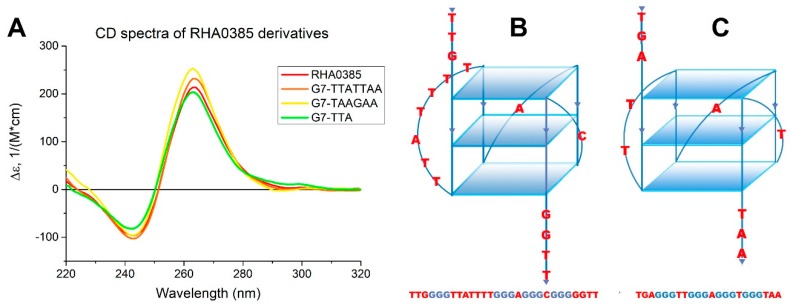
(**A**) The CD spectra of RHA0385 and its derivatives at 20 °C in PBSK have the positive band at 263 nm and negative band at 243 nm, which are characteristic for the parallel G-quadruplex. (**B**) Schematic drawing of putative structure of RHA0385 aptamer as a parallel G-quadruplex with three G-quartets and 7:1:1 loops. The major 7-nucleotide loop has the TTATTTT sequence. (**C**) Schematic drawing of structure of the parallel G-quadruplex 11/23 with 2:1:1 loops, described previously [33].

G-quadruplexes of different topologies can be distinguished by CD spectroscopy. The CD spectrum of RHA0385 at 20 °C in the presence of 10 mM potassium and 140 mM sodium cations has the positive band at 263 nm and negative band at 243 nm (Figure 1A). This CD spectral shape is characteristic to the *syn* conformation of the glycosidic bond of guanosines in G-quartets, i.e., in the parallel G-quadruplexes [41].

The loops of G-quadruplexes usually consist of non-guanine nucleotides between G-blocks that form G-tetrads [42]. Thus, the 7 nucleotides TTATTTT between the first and second G-blocks in RHA0385, as well as the single nucleotides A and C between subsequent G-blocks, are loops (Figure 1B). G-score value of this putative structure calculated by the Quadruplex forming G-Rich Sequences (QGRS) Mapper [43] was highest for the RHA0385 sequence and equal to 36. The topology of RHA0385 was similar to the previously characterized parallel G-quadruplex 11/23 deriving from a promoter sequence [33] (Figure 1C).

To study the role of the major 7-nucleotide loop in the structure and function of aptamer RHA0385, derivatives of this DNA aptamer with an altered loop sequence were proposed. It was previously reported that TAA and GAA were the most abundant patterns in unpaired regions of aptamers to hemagglutinin of influenza A virus [15]. In the RHA0385 loops and hanging ends, these patterns are absent, so they were inserted into the major loop. In the G7-TTATTAA oligonucleotide, the last TT of the loop was altered to AA, which inserted the TAA pattern while retaining the original loop length. The major loop of the G7-TAAGAA oligonucleotide contained two patterns, TAA and GAA, and its length decreased from 7 to 6 nucleotides. In the G7-TTA aptamer, the major loop was shortened from 7 to 3 nucleotides. Therefore, this set of oligonucleotides allowed the study of the effects of both the length and the sequence of the major loop on aptamer structure and affinity. 

### 3.2. Structural Analysis

Structural analysis of all oligonucleotides was performed by circular dichroism (CD) spectroscopy, nuclear magnetic resonance (NMR), and size-exclusion chromatography (SE-HPLC). 

CD spectra of the RHA0385 derivatives are characteristic for parallel G-quadruplexes and are almost the same as for the initial aptamer, except the slight differences in the CD intensities of both negative and positive maxima (Figure 1A). 

The comparison of the denaturation and renaturation profiles of the G-quadruplex structure by CD spectroscopy allows to determine whether the structure is thermodynamically driven or a result of kinetic control. For the equilibrium process, the hysteresis between the denaturation and renaturation curves is not observed [44], and the thermodynamic parameters such as ΔH°, TΔS°, ΔG°_298_, could be calculated on par with T_m_. The thermodynamic parameters can be easily calculated in the approach of the two-state model with one-stage transition, where only folded and unfolded states of the aptamer exist [45]. The two-state processes can be distinguished by the presence of two isodichroic points, where the CD spectra at different temperatures have similar values of molar circular dichroism at similar wavelengths [41].

**Figure 2:**
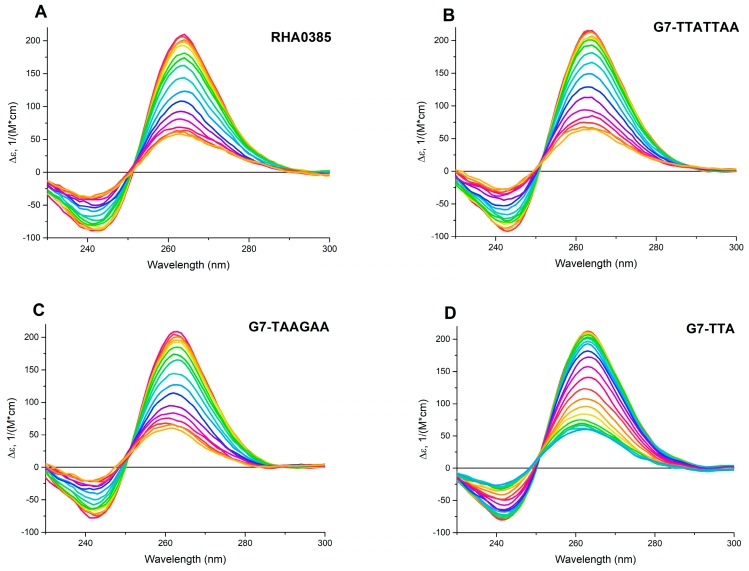
The circular dichroism (CD) spectra of melting of G-quadruplex aptamers from 30 to 70 °C: (**A**) RHA0385, (**B**) G7-TTATTAA, (**C**) G7-TAAGAA; and from 30 to 80 °C: (**D**) G7-TTA. The difference of temperatures between two adjacent spectra was 2.5 °C, the rate of heating was 0.5 °C/min.

**Figure 3:**
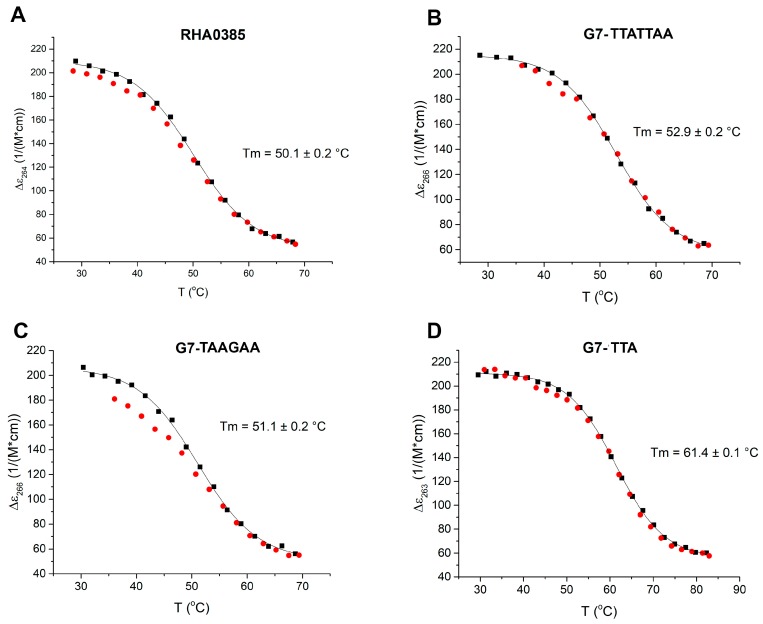
The melting (black dots) and annealing (red dots) curves of (**A**) RHA0385, (**B**) G7-TTATTAA, (**C**) G7-TAAGAA, (**D**) G7-TTA. Melting temperatures are indicated. Only one G-quadruplex (G7-TAAGAA) demonstrates hysteresis under the experimental conditions (2 μM solution in PBSK with overall 10 mM K^+^, with an average heating ramp of 0.5 °C/min).

The CD spectra for both the initial and derived aptamers demonstrated two isodichroic points; therefore, the two-state model can be applied here (Figure 2). The hysteresis during denaturation–renaturation processes can be observed on the plot of thermal dependency of ε value at the positive maximum of the CD spectra (Figure 3). G7-TAAGAA exhibits hysteresis between the melting and annealing processes (Figure 3C); after annealing, the circular dichroism values do not return to the initial level. This could be explained from the conformational polymorphism point of view, so in the case of G7-TAAGAA, the accurate calculation of its thermodynamic parameters cannot be made. For the other aptamers, the thermodynamic parameters were estimated and compared with those for the topologically-related parallel G-quadruplex 11/23 (TGAGGGTTGGGAGGGTGGGTAA) described previously [33] (Table 1).

**Figure 4:**
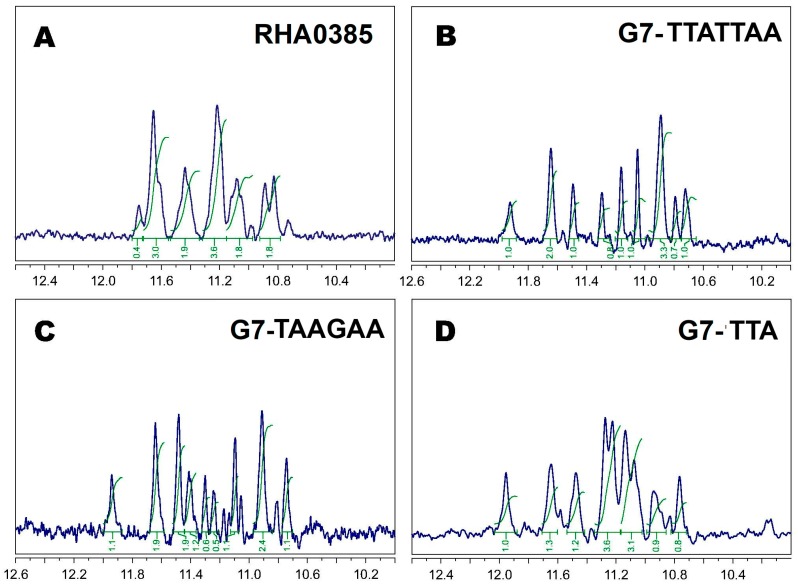
^1^H ‘oligomers filtered’ NMR spectra with ‘*WATERGATE W5*’ water suppression of (**A**) RHA0285, (**B**) G7-TTATTAA, (**C**) G7-TAAGAA, (**D**) G7-TTA at 5 °C in PBSK had the peaks of 12 G-quadruplex imino protons within the range of 10.5–12 ppm; all peaks are numbered in the first approximation for major monomer patterns; all spectra are mathematically processed to suppress a wide oligomeric humps using “multipoint baseline correction” algorithm.

One-dimensional ^1^H NMR spectra analysis of oligonucleotides can be used to characterize their scaffolds. The observed chemical shifts within the range of 10.5–12 ppm (Figure 4) were attributed to the hydrogen-bonded guanine imino protons [46] and unambiguously indicated the presence of G-quartets in the structures of all aptamers studied. The number of peaks in spectra of the G-quadruplex with a single conformation strictly corresponds to the number of guanines participated in the G-quadruplex structure (12 peaks with partially overlapping). Imino-protons regions of ^1^H NMR spectra after baseline correction and ‘oligomers filtered algorithm’ for more concentrated solutions are shown in Figure 4; dilution of aptamers solutions (even up to 20 times) did not lead to significant changes in the NMR spectra patterns. It should be noted that all studied aptamers are very prone to formation of oligomeric patterns at ‘NMR’ concentrations, so an intensive wide oligomeric hump in the imino-protons region of spectra appeared. For a precise baseline correction to suppress ‘mathematically’ a wide oligomeric hump, ‘multipoint baseline correction’ algorithm was applied, which had provided the ‘clean’ spectra in Figure 4.

In our case, some NMR spectra had more than one G-quadruplex pattern (besides oligomers). For example, the spectrum of the initial aptamer, RHA0385, contained widened peaks with fractional areas (see, for example, the peak at 11.8 ppm with area 0.4) due to superposition of several individual spectra (Figure 4A). Additionally, the spectrum of G7-TAAGAA contained more than 12 peaks, and some of them had fractional areas (Figure 4C). Conversely, the NMR spectra of G7-TTATTAA and G7-TTA (Figure 4B,D) each had a set of narrow peaks with partial overlapping, which can be assigned to the 12 imino protons of the 12 guanines in the G-quadruplex. Thus, only two individual aptamers were defined: G7-TTA and G7-TTATTAA (without considering an oligomers formation).

**Figure 5:**
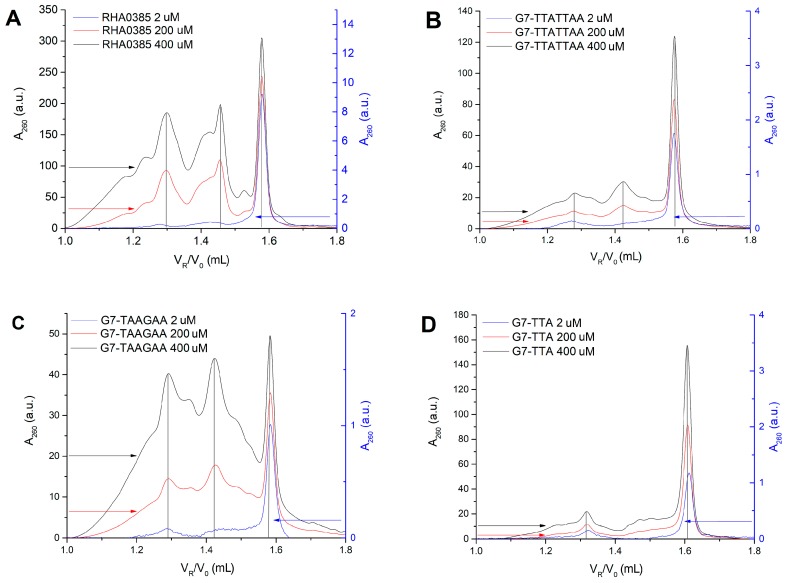
Chromatograms of size-exclusion high performance liquid chromatography SE-HPLC for the 2, 200, 400 μM solutions of (**A**) RHA0385, (**B**) G7-TTATTAA, (**C**) G7-TAAGAA, (**D**) G7-TTA demonstrate different content of mono- and oligomeric forms in solutions of different aptamers. The right Y axis refers to the 2 μM sample, the left one—to 200 and 400 μM. The peaks with 0.9, 1.6, and 2.6 ratios between the expected and the experimental molecular weights are observed and labelled by grey lines.

Parallel G-quadruplexes are known to form dimeric and oligomeric species [47]. Oligonucleotide oligomers can be detected using the SE-HPLC technique developed previously [36,37,48,49]. The oligomeric G-quadruplexes are eluted earlier than the more compact monomeric G-quadruplexes. The peaks with either the 1.6 or 2.6 ratio between the expected and the experimental molecular weights were attributed to the oligomeric conformation, while the peaks with the 0.9 ratio were considered as monomolecular (the relative retention volumes and the quantities of the monomolecular conformations of G-quadruplexes at different concentrations are shown in Supplementary Table S1). In the 400 μM solutions of aptamer RHA0385, three distinct species were observed: mono-, di-, and oligomers with relative retention volumes of approximately 1.58, 1.43, and 1.3 mL respectively. However, the 2 µM solutions predominantly contained monomers (Figure 5A). Aptamer G7-TAAGAA contained even higher proportions of oligomers (Figure 4C), while the G7-TTA (Figure 4D) and G7-TTATTAA (Figure 4B) aptamers were predominantly monomeric even in concentrated solutions. 

In conclusion, alterations in the major loop affected the stability of the scaffold. Two of the newly generated aptamers were shown to be individual G-quadruplexes, contrary to the initial aptamer.

### 3.3. Binding of Aptamers to Recombinant HA and Influenza Viruses

The aK_D_ values of the complexes of RHA0385 with recombinant HA and influenza viruses have been published in our previous work [34]. Here, we performed a more thorough analysis of the data: the aK_D_ values from ELAA experiments were recalculated and the kinetic association and dissociation constants were obtained from SPR curves for RHA0385–HA complexes for the first time. We then compared this aptamer with the three newly designed aptamers (Table 2). 

The study of complex formation between aptamers and recombinant HA was performed with SPR. The proteins were immobilized on a chip surface and the aptamer solutions of different concentrations were used as analytes. The calculated equilibrium dissociation constant (aK_D_) and kinetic association and dissociation constants (k_on_ and k_off_) of each aptamer–HA complex are listed in Table 2. Almost all of these complexes had aK_D_ lower than 100 nM, with the exception of G7-TAAGAA–H7 complex. 

The affinity of each aptamer to the influenza A virus particles was estimated by ELAA analysis. The values of the apparent dissociation constant (aK_D_) were obtained based on aptamer binding to immobilized viruses and are listed in Table 2. These values were smaller than the values for the complexes with recombinant HA. The apparent dissociation constants of complexes with the control virus (influenza B) were all greater than 200 nM.

The newly generated derivative aptamers had similar or higher aK_D_s than the initial aptamer. Both the kinetic association and dissociation constants were slightly worsened with the alterations of the major loop. The exception was G7-TTA, which had a k_off_ 1.2–1.8 times lower than that of the initial aptamer and the lowest aK_D_ of all the newly generated aptamers. On average, the affinity of the aptamers decreased in the following order: RHA0385 > G7-TTA > G7-TTATTAA > G7-TAAGAA. The addition of one or two of the most abundant patterns of hemagglutinin-binding aptamers to the major loop of RHA0385 did not lead to a better affinity, but they also did not abolish the affinity.

### 3.4. Phylogenetic Tree of HA

**Figure 6:**
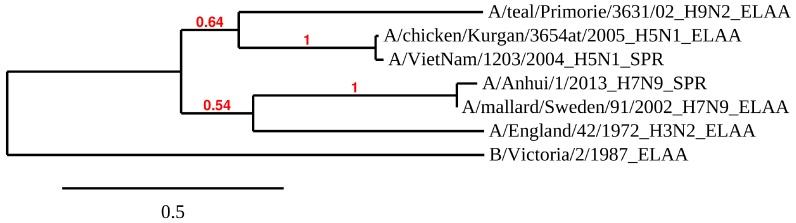
The phylogenetic tree of HA1 subunits of HAs used for the affinity studies of aptamer RHA038 and its derivatives. It was obtained with Phylogeny.fr software [40] from the amino acid sequences of the HA1 subunits of six influenza A and one influenza B viral strains. The scale bar indicates the numbers of amino acid substitutions per site. The bootstrap value is indicated for each inner branch of tree. The method of affinity analysis used for the specific HA was indicated after the full name of the corresponding viral strain.

In order to better understand the difference between the different HAs used for affinity analysis, the phylogenetic tree for the HA1 subunits of six influenza A virus strains and one influenza B strain was constructed (Figure 6). The influenza B virus, which does not bind the aptamers, differs from all influenza A viral strains. Only two sequences for the recombinant HAs, used for our SPR analysis, are known (from H5N1 and H7N9 strains), and there were 4 HAs used in the ELAA studies with whole virus particles. As anticipated, the HA1 subunits of hemagglutinin exhibited high variability in their amino acid sequences. No obvious correlation between aptamer affinity and the distance between the strains in the phylogenetic tree could be found. Thus, understanding how the aptamers recognize will require the identification of the exact binding site of aptamer to HA, and then the amino acids sequences can be compared.

## 4. Discussion

### 4.1. G-Quadruplex Topology of RHA0385

The structural analysis of the RHA0385 aptamer by CD, NMR, and SE-HPLC revealed that RHA0385 folds into a parallel intramolecular G-quadruplex structure in diluted solutions (Figure 1, Figure 4A and Figure 5A). The putative RHA0385 structure, a parallel G-quadruplex with 7:1:1 chain-reversal loops, is shown in Figure 1B. The propeller loops with lengths of one or seven nucleotides are known to exist in a parallel G-quadruplex structure [42]. The seven-nucleotide sequence TTATTTT between the first and second G-strands is referred to as the major loop.

This model is preliminary and needs to be further studied. For example, a shift is possible due to one additional guanine at the 5′- end and two additional guanines at 3′- end of the putative G-quadruplex. A shift of the G-blocks may lead to the coexistence of different conformations and, consequently, to different lengths of the loops. The roles of the 3′- and 5′-ends in aptamer stability and function are not clear and should be studied further. Also, the alteration of loop and hanging ends sequences could affect the ionic sensitivity of an aptamer, so the cationic environment preferred and binding conditions could have changed consequently [50] and this aspect should be studied further.

The putative structure of the aptamer provides the opportunity to make rational alterations in order to better understand its functional determinants. In RHA0385, the major loop seemed likely to be important for aptamer structure and function due to its unusual length and the hypothesis that loops are structured with the G-quadruplex scaffold and act as the binding parts.

### 4.2. The Major Loop Affected G-Quadruplex Stability

Three derivatives of RHA0385 were designed and studied to answer the question about the role of the major loop, TTATTTT, in aptamer structure and affinity: G7-TTATTAA, G7-TAAGAA and G7-TTA. All derivatives retained the topology of a parallel G-quadruplex (Figure 1A and Figure 4). The most stable parallel G-quadruplex was in aptamer G7-TTA, which contains the shortest 3-nucleotide loop. Its free Gibbs energy of folding ΔG°_298_ (−18 kJ/mol) is quite comparable with ΔG°_298_ (−19 kJ/mol) of the previously studied parallel G-quadruplex 11/23 (TGAGGGTTGGGAGGGTGGGTAA) [33], which similarly with G7-TTA possess four G3-blocks and hanging 3′- and 5′-ends but contains a smaller major loop (2 nucleotides). The melting temperatures were nearly identical, although 11/23 has lower entropy and enthalpy changes. Compared to the initial aptamer, RHA0385, the G7-TTA was stabilized due to its favorable entropic summand (−152 ± 2 kJ/mol versus −147 ± 2 kJ/mol), whereas the enthalpy summands were the same (Table 1). The G7-TTATTAA was slightly more stable than the initial RHA0385 (values of ΔG°_298_ are −15 and −13 kJ/mol, respectively) with a simultaneous decrease of both enthalpy and entropy. These data are in agreement with previous studies, where the stability of parallel intramolecular G-quadruplexes can be significantly increased by shortening the loops rather than replacing nucleotide [42,51,52].

### 4.3. The Major Loop Affected the Conformational Profile of the G-Quadruplex

Wide conformational landscape complicates the understanding of structure-activity relationship. It is therefore advisable to study individual conformations, if possible. It is known that parallel G-quadruplexes are inclined to associate into intermolecular complexes [53]. Nevertheless, it is predicted that RHA0385 binds to HA as a monomolecular G-quadruplex because previous affinity studies [23,24] include preformation of the aptamer at low concentrations (1 μM), where the monomolecular conformation dominates for all G-quadruplexes studied (Figure 5). However, SE-HPLC and NMR analyses reveal that RHA0385 and G7-TAAGAA aptamers contained large amounts of oligomeric species in more concentrated solutions. On the contrary, newly generated G7-TTATTAA and G7-TTA aptamers were shown to be individual G-quadruplexes. Thus, these two alterations in the major loop led to a homogeneous conformational profile instead of superposition of several conformations. This peculiarity will be very important considering further practical implementation with large-scale production of aptamers.

### 4.4. The Major Loop Affected the Affinity of the G-Quadruplex Aptamer to Hemagglutinin

**Figure 7:**
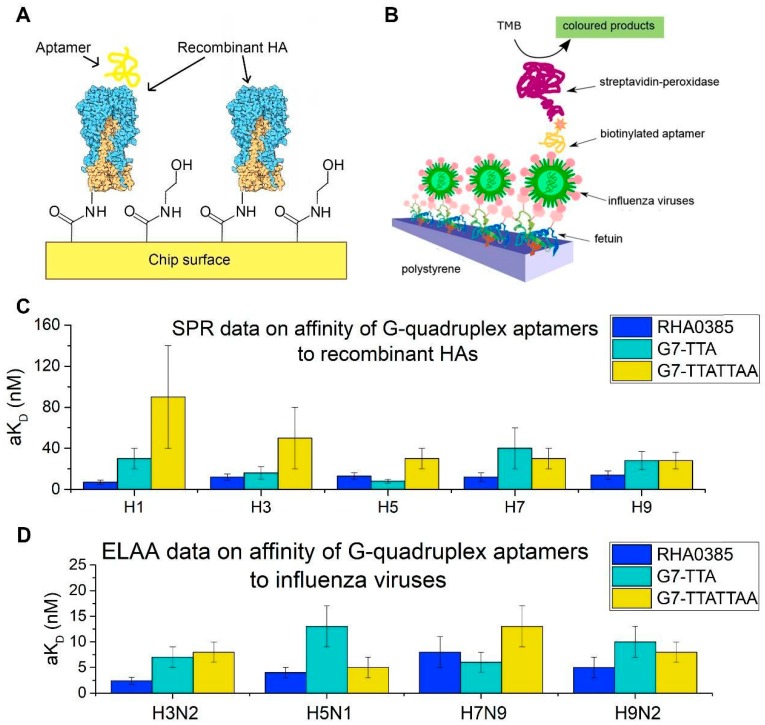
The affinities of the initial aptamer RHA0385 and its derivatives. (**A**) Scheme of SPR analysis of interaction between the aptamer and the recombinant HAs. (**B**) Scheme of ELAA analysis of interaction between the aptamer and the influenza virus particles. (**C**) SPR data on affinities of G-quadruplex aptamers to recombinant HAs from H1, H3, H5, H7, H9 serotypes. (**D**) ELAA data on affinities of G-quadruplex aptamers to the different strains of the influenza A viruses: A/England/42/1972 (H3N2), A/chicken/Kurgan/3654at/2005 (H5N1), A/mallard/Sweden/91/2002 (H7N9), A/duck/Primorie/3628/2002 (H9N2).

To determine the influence of the major loop sequence on affinity, complexes of the two newly generated conformationally homogeneous aptamers with recombinant HA and influenza viruses were studied. The complexes with rHA were studied with SPR (Figure 7A), and ELAA was used for complexes with viral particles (Figure 7B). The equilibrium constants of RHA0385–HA complexes have been described previously [24]. 

In each functional analysis, immobilized protein or virus was used (Figure 7A,B), and the aptamer was in solution. Differences between the protein conditions on the viral surface in the ELAA analysis and those on the surface of the SPR chip may affect the aK_D_ value. Thus, the aK_D_ values can be compared accurately only within the same technique.

Overall, the alterations of the RHA0385 major loop retained or slightly decreased affinity. Even the weakest complexes had aK_D_ values lower than the arbitrary binding threshold of 100 nM. On average, the affinity of aptamers decreased in the following order: RHA0385 > G7-TTA > G7-TTATTAA > G7-TAAGAA. Notably, aptamer G7-TTA had the lowest kinetic dissociation constants that were up to 1.8-fold, lower than that of RHA0385 (Table 2). Thus, G7-TTA formed more stable complexes among other derivatives of RHA0385, although it did not have the highest association rates. It should be noted that the increased stability of G7-TTA aptamer did not lead to increase in binding to HA, probably because the melting temperatures of these G-quadruplexes are higher than the temperature of binding assays (25 °C).

Each aptamer also had different affinities to HA from different strains. HA is known to be a protein with a highly variable sequence [20] (Figure 6). Aptamer RHA0385 had the highest affinity to H1 in SPR (viral strain is not known) and H3 in ELAA (A/England/42/1972 (H3N2)), whereas G7-TTA preferentially bound H5 in SPR (A/Vietnam/1203/2004 (H5N1)) and H7 in ELAA (A/mallard/Sweden/91/2002 (H7N9)). Aptamer G7-TTATTAA had nearly equal affinity to H5, H7, and H9 in SPR (A/Vietnam/1203/2004 (H5N1) and A/Anhui/1/2013 (H7N9); the viral strain of H9 protein is not known) and preferred H5 in ELAA (A/chicken/Kurgan/3654at/2005 (H5N1)). Alterations in the major loop affected the choice of binding partner while retaining high affinity in most cases. The affinity of the aptamers had no correlation with antigenic labelling of HA using antibodies. Even HAs with similar sequences (Figure 6), for instance, HA on surface of the A/chicken/Kurgan/3654at/2005 (H5N1) virus and recombinant HA from A/Vietnam/1203/2004 (H5N1) strain, did not correlate with each other (Figure 7C,D). To further analyze these binding differences, the exact binding sites need to be identified, so the specific amino acids bound by the aptamers in each HA can be compared.

The current data indicate that the major loop is influential but there are no rigorous restrictions in its sequence for the binding of HA. It is likely that the conformation of the parallel G-quadruplex itself recognizes the conserved quaternary HA structure, and the major loop fine-tunes binding increasing the stability and conformational homogeneity of the G-quadruplex aptamer to HA. To find the answer on the questions, which part of G-quadruplex aptamer is responsible for the complex formation and which aptamer modification should enhance the function, extended research is necessary using a set of different viral strains and other RHA0385 derivatives.

## 5. Conclusions

A structural analysis revealed that RHA0385, a DNA aptamer to influenza A virus hemagglutinin, folds into parallel G-quadruplex structure. A putative structure of the aptamer was proposed with 7:1:1—nucleotide loops. The major loop altered in specific ways to determine its role in aptamer structure, stability, and function. It was found that alterations in the major loop have no significant influence on the function of the aptamer and its G-quadruplex nature, but some alterations (such as shortening the loop or adding several purines) stabilized the G-quadruplex core and caused the intramolecular conformations to be more favorable even in concentrated solutions. The role of major loop is probably restricted to the fine-tuning of the binding of the G-quadruplex aptamer to HA. The novel aptamers G7-TTATTAA and G7-TTA retains the functional abilities of the initial aptamer while also having lower conformational diversity and higher thermal and thermodynamic stability.

## Figures and Tables

**Table 1:** Thermodynamic parameters and T_m_ of G-quadruplex aptamers folding. Comparison with parameters of parallel G-quadruplex 11/23 (TGAGGGTTGGGAGGGTGGGTAA) [33] is described further in the Discussion.

Aptamer	ΔH°, kJ/mol	TΔS°, kJ/mol	ΔG°_298_, kJ/mol	T_m_, °C
RHA0385 (TTATTTT loop)	−165 ± 2	−152 ± 2	−13 ± 4	50.1 ± 0.2
G7-TTATTAA	−177 ± 2	−162 ± 2	−15 ± 4	52.9 ± 0.2
G7-TAAGAA	n.c.	n.c.	n.c.	51.1 ± 0.2
G7-TTA	−165 ± 3	−147 ± 2	−18 ± 5	61.4 ± 0.1
11/23 (TT loop) [33]	−173	−154	−19	64

**Table 2:** Apparent dissociation constants aK_D_ and rate constants k_on_ and k_off_ of aptamer–HA complexes, calculated from SPR (with recombinant HA) and ELAA (with viral particles) data. The influenza A viral strains for ELAA analysis are A/England/42/1972 (H3N2), A/chicken/Kurgan/3654at/2005 (H5N1), A/mallard/Sweden/91/2002 (H7N9), A/duck/Primorie/3628/2002 (H9N2).

Method	HA type	Type of Constant	RHA0385	G7-TTATTAA	G7-TAAGAA	G7-TTA
SPR	H1	k_on_, 1 × 10^5^/(M × s)	10 ± 2	1.9 ± 0.2	1.9 ± 0.2	1.2 ± 0.4
		k_off_, 1 × 10^−3^/s	6.9 ± 0.9	17 ± 7	11 ± 4	4.0 ± 0.4
		aK_D_, nM	7 ± 2	90 ± 50	60 ± 30	30 ± 10
	H3	k_on_, 1 × 10^5^/(M × s)	5.1 ± 0.7	2.8 ± 0.3	1.5 ± 0.3	2.8 ± 3.4
		k_off_, 1 × 10^−3^/s	6.1 ± 0.5	14 ± 7	9 ± 1	5 ± 1
		aK_D_, nM	12 ± 3	50 ± 30	60 ± 20	16 ± 6
	H5	k_on_, 1 × 10^5^/(M × s)	5.1 ± 0.6	3.7 ± 0.6	1.4 ± 0.2	2.0 ± 0.3
		k_off_, 1 × 10^−3^/s	6.7 ± 0.8	12 ± 3	8.2 ± 0.7	4.0 ± 0.6
		aK_D_, nM	13 ± 3	30 ± 10	60 ± 10	20 ± 6
	H7	k_on_, 1 × 10^5^/(M × s)	6.1 ± 0.7	6 ± 1	0.9 ± 0.1	1.4 ± 0.4
		k_off_, 1 × 10^−3^/s	8 ± 2	17 ± 4	12 ± 4	5.0 ± 0.8
		aK_D_, nM	12 ± 4	30 ± 10	130 ± 50	40 ± 20
	H9	k_on_, 1 × 10^5^/(M × s)	4.5 ± 0.6	4.5 ± 0.7	1.5 ± 0.3	1.7 ± 0.4
		k_off_, 1 × 10^−3^/s	6.4 ± 0.8	13 ± 2	8 ± 3	4.6 ± 0.5
		aK_D_, nM	14 ± 4	28 ± 8	60 ± 20	28 ± 9
ELAA	H3	aK_D_, nM	2 ± 1	8 ± 2	17 ± 5	7 ± 2
	H5	aK_D_, nM	4 ± 1	5 ± 2	9 ± 3	13 ± 4
	H7	aK_D_, nM	8 ± 3	13 ± 4	13 ± 4	6 ± 2
	H9	aK_D_, nM	5 ± 2	8 ± 2	7 ± 2	10 ± 3

## Supplementary Materials

The following are available online at https://www.mdpi.com/2218-273X/10/1/119/s1, Table S1: The results of SE-HPLC analysis on the monomolecular conformation of G-quadruplex aptamers.
