# Supplementary material for: Structural and Functional Aspects of G-Quadruplex Aptamers Which Bind a Broad Range of Influenza A Viruses
Source: Biomolecules. 2020 Jan 10;10(1):119. doi: 10.3390/biom10010119 (PMC7022617; doi:10.3390/biom10010119)
Supplement: Supplementary file 1 [file biomolecules-10-00119-s001.pdf]

Supplementary Table S1. The results of SE-HPLC analysis on the monomolecular conformation of G-quadruplex aptamers. The V_R/V_0 parameter is a relative retention volume of the peak with 0.9 ratio between expected and experimental molecular weights, which were attributed to the monomolecular conformation. The quantity of the monomolecular form was calculated as the relative peak area.

Aptamer	Concentration, µM	V_R/V_0	Quantity, %
RHA0385	2	1.58	100
	200	1.58	31
	400	1.58	21
G7-TTATTAA	2	1.57	86
	200	1.58	51
	400	1.58	39
G7-TAAGAA	2	1.58	91
	200	1.58	24
	400	1.58	15
G7-TTA	2	1.61	86
	200	1.61	90
	400	1.61	69
